# Are lung ultrasound features more severe in infants with bronchiolitis and coinfections?

**DOI:** 10.3389/fped.2023.1238522

**Published:** 2023-12-15

**Authors:** Domenico Umberto De Rose, Chiara Maddaloni, Ludovica Martini, Sara Ronci, Flaminia Pugnaloni, Gabriella Marrocco, Alessandra Di Pede, Velia Chiara Di Maio, Cristina Russo, Maria Paola Ronchetti, Carlo Federico Perno, Annabella Braguglia, Flaminia Calzolari, Andrea Dotta

**Affiliations:** ^1^ Neonatal Intensive Care Unit, “Bambino Gesù” Children’s Hospital IRCCS, Rome, Italy; ^2^ PhD Course in Microbiology, Immunology, Infectious Diseases, and Transplants (MIMIT), Faculty of Medicine and Surgery, “Tor Vergata” University of Rome, Rome, Italy; ^3^ Neonatal Sub-Intensive Care Unit and Follow-up, “Bambino Gesù” Children’s Hospital IRCCS, Rome, Italy; ^4^ Microbiology and Diagnostic Immunology Unit, “Bambino Gesù” Children’s Hospital IRCCS, Rome, Italy

**Keywords:** bronchiolitis, newborns, LUS, RSV, rhinovirus, viruses, influenza, respiratory infections

## Abstract

**Background:**

The lung ultrasound (LUS) score can be a useful tool to predict the need for respiratory support and the length of hospital stay in infants with bronchiolitis.

**Objective:**

To compare lung ultrasound features in neonates and infants up to three months of age with bronchiolitis to determine whether LUS scores (range 0–36) differ in infants with coinfections or not.

**Methods:**

Neonates and infants younger than three months admitted to neonatal units from October 2022 to March 2023, who underwent lung ultrasound evaluation on admission, were included in this retrospective study.

**Results:**

We included 60 patients who underwent LUS evaluation at admission. Forty-two infants (70.0%) had a single viral infection. Eighteen infants (30.0%) had a coinfection: fifteen infants (25.0%) had more than one virus at PCR; one infant (1.7%) had both a viral coinfection and a viral-bacteria coinfection; two infants (3.3%) had viral-bacteria coinfection. Infants with a single viral infection and those with coinfections had similar LUS scores globally and in different lung zones. An LUS score higher than 8 was identified to significantly predict the need for any respiratory support (*p* = 0.0035), whereas an LUS score higher than 13 was identified to significantly predict the need for mechanical ventilation (*p* = 0.024).

**Conclusion:**

In our small cohort of neonates and infants younger than three months hospitalized with bronchiolitis, we found no statistically significant differences in the LUS score on admission between patients with a single viral infection and those with multiple infections.

## 1. Introduction

Bronchiolitis is a common viral lower respiratory tract infection affecting infants, who require respiratory support and intravenous hydration in most severe cases (1, 2). In particular, infants with pre-existing risk factors (i.e., prematurity, bronchopulmonary dysplasia, congenital heart diseases, immunodeficiency, neuromuscular diseases, cystic fibrosis, and Down syndrome) present a significant risk of severe bronchiolitis and should be carefully assessed (2).

Today, point-of-care lung ultrasound is a proven method for examining infant lung illness since it is simple to use, bedside, consistent, and radiation-free. It can help clinicians to predict prognosis, integrating pulmonary anomalies (lung B-lines, subpleural consolidation, and abnormalities of the pleural line) and clinical findings (3–6). Indeed, the lung ultrasound (LUS) score can be a feasible quantitative method to predict the need for respiratory support and the length of hospital stay (5).

With the spread of multiplex polymerase chain reaction (PCR) in the medical setting, the simultaneous identification of two or more viruses in bronchiolitis and the diagnosis of viral coinfection is becoming more common (7, 8). However, these molecular tests are often available only in III-level hospitals, and it could be difficult to discriminate coinfections in I- and II-level hospitals.

Our hypothesis was that infants with coinfections could have worse LUS scores than patients with a single infection, and this could lead to faster bedside identification of the severity of the clinical picture when a molecular test to discriminate coinfections is not available. Therefore, the aim of our study was to compare lung ultrasound features in neonates and infants up to three months of age with bronchiolitis to determine whether LUS scores differ in infants with coinfections or not.

## 2. Methods

### 2.1. Study design

We retrospectively collected data (gender, gestational age, birthweight, age and weight at admission, need for non-invasive or invasive respiratory support, need for intravenous infusion, need for enteral fasting) from the medical records of neonates and infants aged <3 months, admitted to the Neonatal Intensive Care Unit and Neonatal Sub-Intensive Care Unit of our hospital for bronchiolitis from October 2022 to March 2023.

We excluded infants who did not undergo lung ultrasound evaluation on admission and those with incomplete clinical data. In order to study a homogeneous sample of previously “healthy” infants, we also excluded infants hospitalized only for apnoea (such as preterm infants with negative microbiological tests) and infants with any high-risk conditions for respiratory failure (such as congenital heart disease or pulmonary malformations not already surgically repaired, neurologic disorders, and immunodeficiency).

Hospitalized infants were managed according to the latest guidelines and recent data from the literature (1, 9–12), as previously described (13). In our clinical routine, infants were discharged 24 h after they no longer needed respiratory support, and they achieved full enteral feeding again without the need for intravenous infusion.

### 2.2. Lung ultrasound score

Lung ultrasonography (LUS) was performed on admission by four neonatologists (D.U.D.R., C.M., L.M., and S.R.), who were specifically trained in lung ultrasound and routinely performed lung ultrasound scans for clinical practice in our NICU. They used a pocket-size “iViZ” wireless ultrasound scanner (Fujifilm Sonosite Inc, Bothell, WA—USA) with a linear probe (13 MHz). To minimize neonatal discomfort, LUS assessments were performed exclusively after the patient's routine care, during quiet spontaneous sleep and/or sedation (this last only in the case of mechanically ventilated patients, after obtaining parental consent). We performed sagittal scans in order to explore the whole lungs and to assign the LUS. We evaluated patients in supine and prone positions, considering six regions for each lung (for greater accuracy): two on the anterior side, two on the posterior side, and two laterally. LUS score was assigned at the moment of evaluation giving a score from 0 to 3 to each region as follows: 0 indicates A-pattern (defined by the presence of lung sliding and artifactual horizontal A-lines), 1 indicates B-pattern (defined as the presence of ≥3 vertical B-lines extending from the pleural line, indicating the presence of fluid in the interstitium), 2 indicates a severe B-pattern (defined as the presence of crowded and coalescent B-lines with or without consolidations limited to the subpleural space, indicating fluid in the alveolar space), and 3 indicates extended consolidations, as the presence of a tissue structure with or without hyperechoic punctiform images resembling air bronchograms (14).

### 2.3. Clinical scoring tools

In order to compare the severity of each case at admission, we calculated four clinical severity scores using admission clinical data (13). The Wang Bronchiolitis Severity Score (WBSS) was calculated with four items (respiratory rate, general appearance, wheezing, retractions), each ranging from 0 to 3, except for the general condition (scored only 0 or 3), with a total from 0 to 12 (15). The Kristjansson Respiratory Score (KRS) is based on five signs (respiratory rate, general appearance, wheezing, retractions, and skin color), each from 0 to 2, with a total from 0 to 10 (16). The ReSVinet Scale (ReSVS), recently proposed by Respiratory Syncytial Virus network, is based on seven signs (feeding intolerance, medical intervention, respiratory difficulty, apnea, general condition and fever), each ranging from 0 to 3 points (except for apnea, scored only 0 or 3, and fever, scored from 0 to 2), with a total from 0 to 20 (17). The Global Respiratory Severity Score (GRSS) was calculated only in infants with RSV by entering ten parameters (age, oxygen saturation, respiratory rate, general appearance, wheezing, rhales/ronchi, retractions, skin color, lethargy, and poor air movement) in an interactive tool (available at: https://rprc.urmc.rochester.edu/app/AsPIRES/RSV-GRSS/) (13).

### 2.4. Outcomes

The primary outcome was to compare the LUS scores between infants with bronchiolitis with a single viral infection and those with a viral or bacterial coinfection (intended as the simultaneous identification of more than one virus/bacterium on the PCR from nasopharyngeal swabs).

The secondary outcome was to evaluate if the LUS score at admission predicted the need for any respiratory support and the need for mechanical ventilation in infants with bronchiolitis.

We also compared clinical severity scoring tools and clinical features (i.e., need for respiratory support, need for intensive care unit admission, length of respiratory support, and length of hospital stay) between the two groups in order to rule out any differences not related to pathogens.

### 2.5. Microbiology testing

All patients enrolled were studied with nasopharyngeal swabs for the identification of respiratory viruses (Influenza virus, Respiratory syncytial virus, Adenovirus, Enterovirus, Parainfluenza virus, Metapneumovirus, Bocavirus, Rhinovirus, and Coronaviruses, including NL63/229E/OC43 and SARS-CoV-2). The nasopharyngeal swabs were analyzed by the multiplex real-time polymerase chain reaction (RT-PCR) “AllplexTM Respiratory Panel Assays” on the All-in-One Platform (Seegene, Korea), as previously described (18). A standard culture of bronchoalveolar lavage fluid (as bronchial secretions from the endotracheal tube) was also performed in infants who underwent mechanical ventilation.

### 2.6. Ethical statement

The authors assert that all procedures of the study comply with the ethical standards of the institutional and national research committee and with the 1964 Helsinki Declaration and its later amendments or comparable ethical standards (19). Personal data were restricted to essential information and were treated in order to guarantee the respect of the privacy of the involved patients, as specifically stated by Italian Law D. Lgs. n.196 of 2003 about personal data protection. Written informed consent was not required, as the study is retrospective with no patient-identifiable information. Despite this, our Scientific Directorate validated the study before the submission to the journal, as in our hospital, all studies performed have to be approved by this office.

### 2.7. Statistical analysis

Data are presented as numbers and percentages for categorical variables for statistical analyses. Continuous variables are expressed as mean ± standard deviation (SD) if normally distributed or as the median and interquartile range (IQR) if normality cannot be accepted. Data distribution was evaluated by the Shapiro–Wilk test. Comparisons between groups were made with Fisher's test, *t*-test or Mann–Whitney test as appropriate. By the receiver operating characteristic (ROC) analysis, the area under the ROC curve (AUC) and Youden's index (best cut-off point) were used to evaluate the ability of the single tool to predict the need for respiratory support. A *p*-value < 0.05 was considered statistically significant. Data were analyzed with the MedCalc Software package for Windows, release 12.7 (MedCalc Software, Belgium).

## 3. Results

### 3.1. Patients

From 1st October 2022 to 31st March 2023, we admitted 146 neonates and infants with acute bronchiolitis. Eighty-six infants were excluded because of the lack of lung ultrasonography score evaluation on admission (80 infants), because of congenital malformations (4 infants: one with ventricular septal defect, one with ventricular septal defect and patent ductus arteriosus, one with scimitar syndrome and right pulmonary hypoplasia and one with atrioventricular septal defect), and because of bronchopulmonary dysplasia (2 infants). Therefore, we included 60 infants in this study (Table 1). Of them, forty-two infants (70.0%) had a single viral infection. Eighteen infants (30.0%) had a coinfection: fifteen infants (25.0%) had more than one virus at PCR; one infant (1.7%) had both a viral coinfection and a viral-bacteria coinfection (RSV, Coronavirus 229E, *Haemophilus influenzae*); two infants (3.3%) had viral-bacteria coinfection (one infant with RSV, *Streptococcus pneumoniae* and *Haemophilus influenzae*, and one infant with RSV and *Bordetella parapertussis*). Table 2 shows viruses causing bronchiolitis in included patients.

**Table 1:** Clinical characteristics and procedures of included patients with acute bronchiolitis.

	Patients (*n* = 60)	Patients with a single viral infection (*n* = 42)	Patients with coinfections (*n* = 18)	*p*-value
Clinical characteristics				
Males, *n* (%)	32 (53.3%)	25 (59.5%)	7 (38.9%)	0.168
Median gestational age, weeks (IQR)	38 (38–39)	38 (38–39)	38 (37–39)	0.087
Median birthweight, grams (IQR)	3200 (2785–3450)	3200 (2930–3480)	3205 (2560–3430)	0.438
Median age at admission, days	26 (18–41)	23 (17–47)	29 (22–36)	0.298
Median WBSS at admission	6.5 (4.0–8.0)	7.0 (4.0–8.0)	6.0 (4.0–8.0)	0.955
Median KRS at admission	4.0 (3.0–6.0)	4.0 (3.0–6.0)	4.0 (3.0–7.0)	0.341
Median RSVS at admission	9.5 (8.0–12.0)	10.0 (8.0–12.0)	8.5 (6.0–12.0)	0.350
Median GRSS at admission (for RSV infants only)	6.25 (4.51–7.40)	6.32 (4.47–7.32)	6.22 (4.51–7.49)	0.986
Procedures				
Need for non-invasive respiratory support, *n* (%)	54 (90.0%)	40 (95.2%)	14 (77.8%)	0.060
Need for supplemental oxygen >21%, *n* (%)	39 (65.0%)	28 (66.7%)	11 (61.1%)	0.771
Need for invasive ventilation, *n* (%)	3 (5.0%)	1 (2.4%)	2 (11.1%)	0.212
Need for intravenous infusion, *n* (%)	56 (93.3%)	39 (92.9%)	17 (94.4%)	1.000
Need for enteral fasting, *n* (%)	12 (20.0%)	9 (21.4%)	3 (16.7%)	1.000

**Table 2:** Identified viruses causing bronchiolitis in this cohort.

	Patients (*n* = 60)
Respiratory syncytial virus	54 (90.0%)
Rhinovirus	13 (21.7%)
Influenza A	3 (5.0%)
Metapneumovirus	1 (1.7%)
Parainfluenza 3 virus	1 (1.7%)
Coronavirus OC43	2 (3.3%)
Coronavirus 229E	1 (1.7%)

The two groups of infants with a single viral infection and those with coinfections were similar in clinical characteristics and the need for procedures, as shown in Table 1. Furthermore, we found no significant differences in the four clinical severity scores between patients with a single viral infection and those with coinfections. Thirty-five patients (58.3%) were neonates, whereas 25 (41.7%) were within three months of life. Three patients (5.0%) were born preterm (range: 26–35 weeks of gestational age). Among 54 infants who required respiratory support, 39 infants/60 (65.0%) received high-flow nasal cannula (HFNC) as maximum support, 5/60 (8.3%) received nasal continuous positive airway pressure (nCPAP), 7/60 (11.7%) received nasal intermittent positive pressure ventilation (nIPPV), one (1.7%) received conventional mechanical ventilation (CMV) and two (3.3%) received high-frequency oscillatory ventilation (HFOV).

The median length of stay was 6 days (IQR 4–8.3) and it was similar between infants with a single viral infection and in those with coinfections: 6.0 days (IQR 4.0–9.0) vs. 5.0 days (IQR 4.0–8.0) (*p* = 0.406).

The median length of respiratory support was 5 days (IQR 3–7) and it was similar between infants with a single viral infection and in those with coinfections: 5.0 days (IQR 3.0–7.0) vs. 4.5 days (IQR 3.0–7.0) (*p* = 0.818). The three infants who underwent mechanical ventilation required a median of 9 days of invasive respiratory support (IQR 6–9) and a further median of 6 days of non-invasive respiratory support (IQR 6–7).

### 3.2. Lung ultrasound features

Infants who needed respiratory support (HFNC, nCPAP, nIPPV or mechanical ventilation) were initially identified by significantly higher LUS scores rather than infants who underwent no respiratory support: 12.0 (IQR 10.0–15.0) vs. 7.5 (IQR 4.0–11.0) (*p* = 0.041). Similarly, infants who required invasive ventilation presented a trend towards higher LUS scores rather than infants who required non-invasive respiratory support only, although not significantly: 17.0 (IQR 15.5–18.5) vs. 12.0 (IQR 10.0–15.0) (*p* = 0.064).

**Figure 1:**
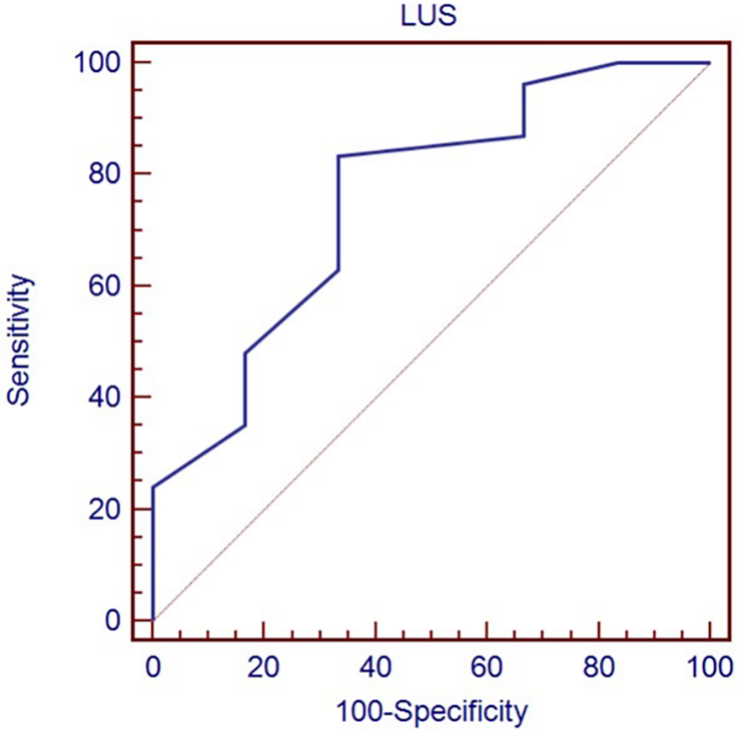
Receiver-operating characteristic curve of LUS score in predicting the need for any respiratory support.

**Figure 2:**
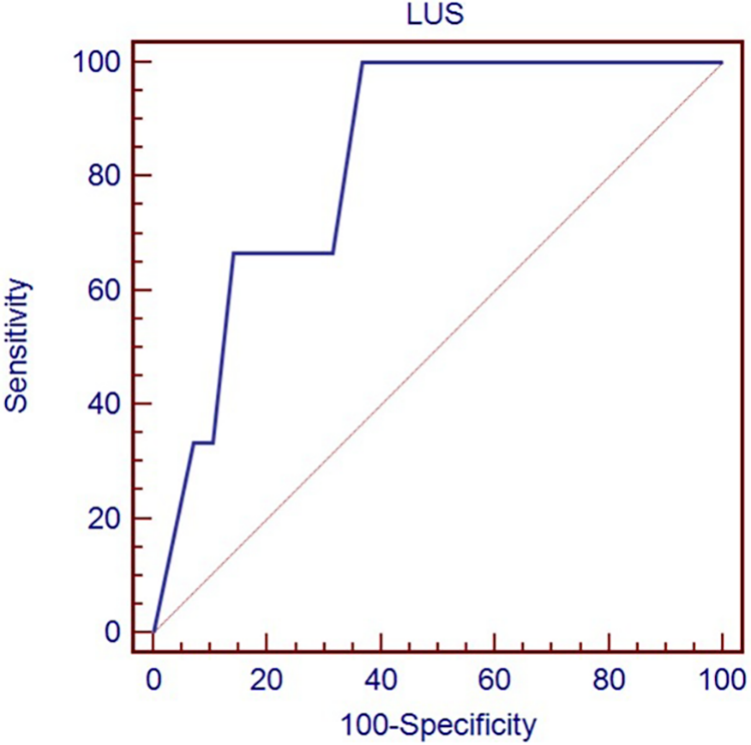
Receiver-operating characteristic curve of LUS score in predicting the need for mechanical ventilation.

Table 3 shows the optimal cut-off of LUS (>8) in predicting the need for any respiratory support (*p* = 0.0035), identified by the ROC curve (Figure 1), and the optimal cut-off of LUS (>13) in predicting the need for mechanical ventilation (*p* = 0.024), identified by the ROC curve (Figure 2).

**Table 3:** LUS score ability in predicting the need for respiratory support.

	Optimal cut-off of LUS score	Area under the ROC curve (AUC)	Standard error	95% confidence interval	Sensitivity (95% CI)	Specificity (95% CI)
Need for any respiratory support	>8	0.756	0.088	0.628–0.858	83.3 (70.7–92.1)	66.7 (22.7–94.7)
Need for mechanical ventilation	>13	0.833	0.147	0.715–0.917	100.0 (30.5–100.0)	63.2 (49.3–75.5)

Infants with a single viral infection and infants with coinfections had similar LUS scores, both globally and in individual lung zones (Table 4).

**Table 4:** LUS scores in different lung zones.

	Patients (*n* = 60)	Patients with a single viral infection (*n* = 42)	Patients with coinfections (*n* = 18)	*p*-value
Total LUS score	12.0 (9.0–15.0)	12.5 (10.0–15.0)	11.5 (7.0–15.0)	0.337
LUS*all right zones	6.0 (4.8–8.0)	6.0 (4.0–8.0)	6.0 (5.0–8.0)	0.759
LUS*right upper anterior	1.0 (0.0–1.0)	1.0 (0.0–1.0)	1.0 (0.0–1.0)	0.567
LUS*right lower anterior	1.0 (1.0–1.0)	1.0 (1.0–1.0)	1.0 (1.0–1.0)	0.572
LUS*right upper lateral	1.0 (0.0–1.0)	1.0 (0.0–1.0)	1.0 (0.0–1.0)	0.891
LUS*right lower lateral	1.0 (1.0–2.0)	1.0 (1.0–2.0)	1.0 (1.0–2.0)	0.872
LUS*right upper posterior	1.0 (1.0–1.0)	1.0 (1.0–1.0)	1.0 (1.0–2.0)	0.949
LUS*right lower posterior	1.0 (1.0–2.0)	1.0 (1.0–2.0)	1.0 (1.0–2.0)	0.321
LUS*all left zones	6.0 (5.0–8.0)	6.0 (5.0–8.0)	6.0 (2.0–7.0)	0.147
LUS*left upper anterior	1.0 (0.0–1.0)	1.0 (0.0–1.0)	1.0 (0.0–1.0)	0.648
LUS*left lower anterior	1.0 (1.0–1.0)	1.0 (1.0–1.0)	1.0 (0.0–1.0)	0.366
LUS*left upper lateral	1.0 (0.0–1.0)	1.0 (0.0–1.0)	1.0 (0.0–1.0)	0.313
LUS*left lower lateral	1.0 (1.0–1.0)	1.0 (1.0–1.0)	1.0 (1.0–1.0)	0.589
LUS*left upper posterior	1.0 (1.0–1.0)	1.0 (1.0–2.0)	1.0 (0.8–1.0)	0.252
LUS*left lower posterior	1.0 (1.0–2.0)	1.0 (1.0–2.0)	1.0 (1.0–1.0)	0.055

## 4. Discussion

This study is the first to compare the lung ultrasound features in neonates and small infants with a single viral infection and in those with coinfections.

Our incidence of viral coinfection (16/60: 26.7%) was similar to that available in different studies (5%–41%) (18, 20–22), and infants with multiple viral coinfections had no influence on the clinical severity of the disease, as previously described (23). However, considering that the literature reports conflicting data regarding the role of coinfections in acute bronchiolitis (18, 20–22), and that they can have a great clinical impact on the guidelines for the isolation of hospitalized patients, their role should be further studied.

RSV is responsible for 34 million new cases of lower respiratory tract infections and 2.4 million hospitalizations of infants all over the world, with 199,000 deaths per year, mostly in developing countries, and it's easy to identify using antigenic tests (24). Conversely, the diagnosis of co-infections is based on new molecular detection methods such as qualitative PCR (which are only available in some hospitals, but not in first-level ones and in developing countries).

The aim of this study was to verify if lung ultrasound could identify infants with coinfections or not in a cheaper way than molecular methods in hospitals where these lasts are not available, considering our initial hypothesis that patients with coinfections could have a worse LUS pattern than patients with a single infection. However, we found no statistically significant differences in the LUS score on admission between patients with a single viral infection and those with multiple infections.

Conversely, our data confirm the utility of lung ultrasound in the evaluation of infants with bronchiolitis and support the hypothesis that the LUS score, evaluated on admission, can identify those patients who will need respiratory support, as previously reported by different studies (25). In particular, a LUS score above 8 could identify infants who will need respiratory support, whereas we found a higher score (>13) in those who underwent mechanical ventilation. Similarly, Gori et al. identified in a multicenter study that a value of >9 points was the best cut-off value to discriminate between mild bronchiolitis and moderate/severe bronchiolitis (26).

For the first time, in this manuscript, we showed how infants with a single viral infection and infants with coinfections had similar LUS scores, both globally and in different lung zones. Conversely, Ferro et al. previously found a significant difference in the incidence of atelectasis at chest x-ray (18.6% in single viral infection vs. 41.7% in multiple viral infections, *p* = 0.04), but no differences in air trapping or consolidation or other clinical features (18).

Infection by a first virus could enhance or reduce infection and replication of a second virus, resulting in positive (additive or synergistic) or negative (antagonistic) interaction (27). Therefore, more than the number of viruses, their type and interactions play a role in the possible modulation of the damage recognizable by lung ultrasound. Additionally, in some cases, the qualitative PCR result continues to be positive even after the symptomatic infection has resolved, leading us to believe that some cases may not be coinfections but rather the presence of a prior infection. This could provide another potential reason for the absence of clinical differences between the two groups.

Concerning viral-bacterial coinfection, only three patients had a viral-bacterial coinfection. Of them, two infants (one with RSV, Coronavirus 229E, and *Haemophilus influenzae*, and one with RSV, *Streptococcus pneumoniae* and *Haemophilus influenzae*) required mechanical ventilation and presented with high LUS scores (20 and 17, respectively). This supports the hypothesis that bacterial superinfections during RSV bronchiolitis are a risk factor for a more severe clinical picture (28), although the small and heterogeneous sample size included in our study prevents us from drawing strong conclusions.

This study has four main limitations. First, LUS scores were retrospectively collected in infants who have been hospitalized because of bronchiolitis, analyzing medical records in a single center and thus not including infants with milder forms. A *post-hoc* power analysis of our study found a 14.3% power (with a two-sided alpha at *α *=* *0.05), and thus the lack of observed differences in lung ultrasound scores between the two groups may be due to the small sample size. To reach an 80% power, we would have had to enroll at least 227 patients per group; a prospective multicenter study is therefore needed to reach such a number of patients with bronchiolitis. Second, a different lung division in fewer areas (i.e., giving the score only to the anterolateral areas and paravertebral/posterior areas) has been previously used by other authors, with consequently different total scores in comparison to our one (26, 29). Third, in the context of the evaluation of the LUS score's ability to predict the need for respiratory support, the reader should be aware that in our context, patients with persistent oxygen saturation levels below 92% and signs of respiratory distress (tachypnoea, chest retractions, etc.) or respiratory acidaemia on the venous blood gas analysis undergo HFNC as primary respiratory support (starting with 4 L/min up to 10 L/min), considering the lower treatment failure in the group receiving high-flow oxygen therapy in a multicenter randomized controlled trial by Franklin et al. (12). This could have influenced results in infants with a milder disease, who probably needed only low-flow oxygen therapy. However, there were no significant differences in clinical scoring tools (WBSS, KRS, RSVS, or GRSS) between infants with a single viral infection and those with multiple infections, and therefore, the infants included in the two groups had the same clinical severity at admission. Fourth, inter-observer reliability has not been evaluated.

Finally, the nasopharyngeal swab is a reliable “proxy” for the possible presence of the virus in the lower tracts, which are the “best habitat” for Parainfluenza virus 3, Metapneumovirus, and Influenza virus, but not for Rhinoviruses (which represent the most frequent co-infection in this cohort of patients), as well as for Coronaviruses (OC43 and 229E) (30). Indeed, these last have an optimum of growth at the cool temperatures found in the nasal cavity (33–35°C) than at core body temperature (37°C) (31), and thus they may not be present in replicating form in the lower airways unless the patient is subjected to invasive ventilation (in this case, Rhinoviruses are the first cause of viral pneumonia) (32).

Further studies are needed to assess whether which LUS score obtained at admission in these infants with bronchiolitis could predict the best strategy during the hospital stay and if changes in the LUS score could appropriately downgrade the intensity of respiratory support.

## 5. Conclusion

In our small cohort of neonates and infants younger than three months hospitalized with bronchiolitis, we found no statistically significant differences in the LUS score on admission between patients with a single viral infection and those with multiple infections.

However, a score above 8 could identify infants who will need respiratory support, whereas we found a higher score (>13) in those who underwent mechanical ventilation.

## Data Availability

The original contributions presented in the study are included in the article/Supplementary Material, further inquiries can be directed to the corresponding author.

## References

[1] Baraldi E Lanari M Manzoni P Rossi GA Vandini S Rimini A Inter-society consensus document on treatment and prevention of bronchiolitis in newborns and infants. Ital J Pediatr. (2014) 40:1–13. 10.1186/1824-7288-40-65 25344148 PMC4364570

[2] Manti S Staiano A Orfeo L Midulla F Marseglia GL Ghizzi C Update—2022 Italian guidelines on the management of bronchiolitis in infants. Ital J Pediatr. (2023) 49:1–18. 10.1186/s13052-022-01392-6 36765418 PMC9912214

[3] Supino MC Buonsenso D Scateni S Scialanga B Mesturino MA Bock C Point-of-care lung ultrasound in infants with bronchiolitis in the pediatric emergency department: a prospective study. Eur J Pediatr. (2019) 178:623–32. 10.1007/s00431-019-03335-6 30747262

[4] Özkaya AK Yilmaz HL Kendir ÖT Gökay SS Eyüboǧlu İ. Lung ultrasound findings and bronchiolitis ultrasound score for predicting hospital admission in children with acute bronchiolitis. Pediatr Emerg Care. (2020) 36:E135–42. 10.1097/PEC.0000000000001705 30601352

[5] La Regina DP Bloise S Pepino D Iovine E Laudisa M Cristiani L Lung ultrasound in bronchiolitis. Pediatr Pulmonol. (2021) 56:234–9. 10.1002/ppul.25156 33151023

[6] Bobillo-Perez S Sorribes C Gebellí P Lledó N Castilla M Ramon M Lung ultrasound to predict pediatric intensive care admission in infants with bronchiolitis (LUSBRO study). Eur J Pediatr. (2021) 180:2073. 10.1007/s00431-021-04003-4 33638717

[7] Petat H Gajdos V Angoulvant F Vidalain PO Corbet S Marguet C High frequency of viral co-detections in acute bronchiolitis. Viruses. (2021) 13:1–6. 10.3390/v13060990 PMC8229544 34073414

[8] Stobbelaar K Mangodt TC Van der Gucht W Delhaise L Andries J Gille V Risk factors associated with severe RSV infection in infants: what is the role of viral co-infections? Microbiol Spectr. (2023) 1(3):e0436822. 10.1128/spectrum.04368-22 PMC10269756 37212711

[9] Ralston SL Lieberthal AS Meissner HC Alverson BK Baley JE Gadomski AM Clinical practice guideline: the diagnosis, management, and prevention of bronchiolitis. Pediatrics. (2014) 134:e1474–502. 10.1542/peds.2014-2742 25349312

[10] Bresesti I Lista G. Respiratory support of neonate affected by bronchiolitis in neonatal intensive care unit. Am J Perinatol. (2020) 37:S10–3. 10.1055/s-0040-1713604 32898876

[11] Jat KR Mathew JL. Continuous positive airway pressure (CPAP) for acute bronchiolitis in children. Cochrane Database Syst Rev. (2019) 1:CD010473. 10.1002/14651858.CD010473.pub3 30701528 PMC6354031

[12] Franklin D Babl FE Schlapbach LJ Oakley E Craig S Neutze J A randomized trial of high-flow oxygen therapy in infants with bronchiolitis. N Engl J Med. (2018) 378:1121–31. 10.1056/nejmoa1714855 29562151

[13] De Rose DU Maddaloni C Martini L Braguglia A Dotta A Auriti C. Comparison of three clinical scoring tools for bronchiolitis to predict the need for respiratory support and length of stay in neonates and infants up to three months of age. Front Pediatr. (2023) 11:1–7. 10.3389/fped.2023.1040354 PMC9983816 36873647

[14] Brat R Yousef N Klifa R Reynaud S Shankar Aguilera S De Luca D. Lung ultrasonography score to evaluate oxygenation and surfactant need in neonates treated with continuous positive airway pressure. JAMA Pediatr. (2015) 169:e151797. 10.1001/jamapediatrics.2015.1797 26237465

[15] Wang EEL Milner RA Navas L Maj H. Observer agreement for respiratory signs and oximetry in infants hospitalized with lower respiratory infections. Am Rev Respir Dis. (1992) 145:106–9. 10.1164/ajrccm/145.1.106 1731571

[16] Pinto F R Correia-Costa L Azevedo I. Comparison of Kristjansson respiratory score and Wang respiratory score in infants with bronchiolitis in a hospital emergency department. Hong Kong Physiother J. (2020) 40:145–53. 10.1142/S1013702520500146 33005078 PMC7526056

[17] Justicia-Grande AJ Pardo-Seco J Cebey-López M Vilanova-Trillo L Gómez-Carballa A Rivero-Calle I Development and validation of a new clinical scale for infants with acute respiratory infection: the ReSVinet scale. PLoS One. (2016) 11:1–15. 10.1371/journal.pone.0157665 PMC4915666 27327497

[18] Ferro V Boccuzzi E Battaglia M Rossi FP Olita C Giglioni E The role of viral coinfection in bronchiolitis treated with high-flow nasal cannula at pediatric emergency department during 2 consecutive seasons: an observational study. Pediatr Infect Dis J. (2020) 39:102–7. 10.1097/INF.0000000000002512 31725117

[19] Association WM. World medical association declaration of Helsinki: ethical principles for medical research involving human subjects. JAMA. (2013) 310:2191–4. 10.1001/jama.2013.281053 24141714

[20] Brand HK De Groot R Galama JMD Brouwer ML Teuwen K Hermans PWM Infection with multiple viruses is not associated with increased disease severity in children with bronchiolitis. Pediatr Pulmonol. (2012) 47:393–400. 10.1002/ppul.21552 21901859 PMC7168072

[21] Marguet C Lubrano M Gueudin M Le Roux P Deschildre A Forget C In very young infants severity of acute bronchiolitis depends on carried viruses. PLoS One. (2009) 4:e4596. 10.1371/journal.pone.0004596 19240806 PMC2644758

[22] Richard N Komurian-Pradel F Javouhey E Perret M Rajoharison A Bagnaud A The impact of dual viral infection in infants admitted to a pediatric intensive care unit associated with severe bronchiolitis. Pediatr Infect Dis J. (2008) 27:213–7. 10.1097/INF.0b013e31815b4935 18277932

[23] Petrarca L Nenna R Frassanito A Pierangeli A Leonardi S Scagnolari C Acute bronchiolitis: influence of viral co-infection in infants hospitalized over 12 consecutive epidemic seasons. J Med Virol. (2018) 90:631–8. 10.1002/jmv.24994 29226974 PMC7166564

[24] Nair H Simões EAF Rudan I Gessner BD Azziz-Baumgartner E Zhang JSF Global and regional burden of hospital admissions for severe acute lower respiratory infections in young children in 2010: a systematic analysis. Lancet. (2013) 381:1380–90. 10.1016/S0140-6736(12)61901-1 23369797 PMC3986472

[25] Kogias C Prountzos S Alexopoulou E Douros K. Lung ultrasound systematic review shows its prognostic and diagnostic role in acute viral bronchiolitis. Acta Paediatr. (2023) 112:222–32. 10.1111/apa.16578 36261915

[26] Gori L Amendolea A Buonsenso D Salvadori S Supino MC Musolino AM Prognostic role of lung ultrasound in children with bronchiolitis: multicentric prospective study. J Clin Med. (2022) 11:1–15. 10.3390/jcm11144233 PMC9316238 35887997

[27] Piret J Boivin G. Viral interference between respiratory viruses. Emerg Infect Dis. (2022) 28:273–81. 10.3201/eid2802.211727 35075991 PMC8798701

[28] Geoghegan S Erviti A Caballero MT Vallone F Zanone SM Losada JV Mortality due to respiratory syncytial virus burden and risk factors. Am J Respir Crit Care Med. (2017) 195:96–103. 10.1164/rccm.201603-0658OC 27331632

[29] Di Mauro A Cappiello AR Ammirabile A Abbondanza N Bianchi FP Tafuri S Lung ultrasound and clinical progression of acute bronchiolitis: a prospective observational single-center study. Medicina (B Aires). (2020) 56:1–8. 10.3390/medicina56060314 PMC7353897 32604769

[30] Colagrossi L Mattana G Piccioni L Cento V Perno CF. Viral respiratory infections: new tools for a rapid diagnosis. Semin Respir Crit Care Med. (2021) 42:747–58. 10.1055/s-0041-1739306 34918318

[31] Foxman EF Storer JA Fitzgerald ME Wasik BR Hou L Zhao H Temperature-dependent innate defense against the common cold virus limits viral replication at warm temperature in mouse airway cells. Proc Natl Acad Sci U S A. (2015) 112:827–32. 10.1073/pnas.1411030112 25561542 PMC4311828

[32] El-Nawawy A Antonios MAM Meheissen MA Fahim MM. Respiratory viruses associated with severe mechanically ventilated pneumonia in children. J Med Virol. (2022) 94:461–8. 10.1002/jmv.27284 34415627 PMC8426888

